# Human deleterious mutation rate implies high fitness variance, with declining mean fitness compensated by rarer beneficial mutations of larger effect

**DOI:** 10.1101/2023.09.01.555871

**Published:** 2023-09-04

**Authors:** Joseph Matheson, Jason Bertram, Joanna Masel

**Affiliations:** 1 Department of Ecology and Evolutionary Biology, University of Arizona, Tucson, AZ, 85721, USA; 2 Department of Ecology, Behavior, and Evolution, University of California San Diego, San Diego, CA, 92093, USA; 3 Department of Mathematics, University of Western Ontario, London ON, Canada

**Keywords:** mutation load, Muller’s ratchet, Ohta’s ratchet, chromosome number, background selection, genetic hitchhiking

## Abstract

Each new human has an expected *U*_*d*_ = 2 – 10 new deleterious mutations. This deluge of deleterious mutations cannot all be purged, and therefore accumulate in a declining fitness ratchet. Using a novel simulation framework designed to efficiently handle genome-wide linkage disequilibria across many segregating sites, we find that rarer, beneficial mutations of larger effect are sufficient to compensate fitness declines due to the fixation of many slightly deleterious mutations. Drift barrier theory posits a similar asymmetric pattern of fixations to explain ratcheting genome size and complexity, but in our theory, the cause is *U*_*d*_ > 1 rather than small population size. In our simulations, *U*_*d*_ ~2 – 10 generates high within-population variance in relative fitness; two individuals will typically differ in fitness by 15–40%. *U*_*d*_ ~2 – 10 also slows net adaptation by ~13%−39%. Surprisingly, fixation rates are more sensitive to changes in the beneficial than the deleterious mutation rate, e.g. a 10% increase in overall mutation rate leads to faster adaptation; this puts to rest dysgenic fears about increasing mutation rates due to rising paternal age.

## Introduction

The average human begins life with upwards of a hundred new mutations not found in their parents (Lynch, 2010b). Lesecque et al. (2012) assumed that mutations are deleterious only in the 55% of the 6×10^9^ diploid genome that is not dominated by transposable elements, which evolves due to this constraint at 94.3% of the rate, at a point mutation rate of 1.1×10^−8^; this yields an estimated rate of deleterious mutations of 0.55×6×10^9^×0.057×1.1×10^−8^ = 2.1 per replication. This estimate is conservative: some mutations to transposable element regions are deleterious, more recent estimates of the human point mutation rate are slightly higher at ~1.25×10^−8^ (Awadalla et al., 2010; Roach et al., 2010), and non-point mutations and beneficial mutations are neglected. Some therefore argue that the deleterious mutation rate is as high as 10 (Kondrashov, 2017). Mutation rates of this order are not unique to humans (Haag-Liautard et al., 2007; Popovic et al., 2023).

Given such an extraordinarily high deleterious mutation rate, geneticists have long worried about the effects of the resulting “mutation load” on human health (Crow, 1997; Lynch, 2016; Muller, 1950; Vy et al., 2021). Classical infinite sites population genetics theory in the absence of epistasis or linkage disequilibrium predicts that segregating deleterious mutations reduce fitness from 1 (maximum relative fitness for a mutationless individual) to $e^{-U_{d}}$, which means that human fitness is reduced to only 13% of what it could be without deleterious mutations (Haldane, 1937). Matters are worse when we consider the possibility that deleterious mutations might fix. Since removing a single deleterious mutation requires on average one ‘selective death’(Matheson et al., 2023), selection cannot keep up with mutation for deleterious mutation rates above one, resulting in the progressive accumulation (“Ohta’s ratchet” (Ruan et al., 2020)) of slightly deleterious fixations, even in sexual populations.

Partial solutions have been proposed to the puzzle of how populations such as humans persist in the face of such high deleterious mutation rates. Firstly, mutation load is sometimes defined as $L=W_{\text{max}}-\bar{W}$, where *W*_*max*_ represents the fitness of a completely mutationless individual (Haldane, 1937). Since this hypothetical deleterious-mutation-free individual has almost certainly never existed, mutation load concerns depend on assumptions about this hypothetical individual’s fitness (Agrawal & Whitlock, 2012). If a mutation-free human could average a hundred offspring, then reducing human fitness to 13% of that optimum would pose no threat. However, this does not resolve the issue of the progressive accumulation of load.

Secondly, some load presumably affects intrinsically relative fitness traits, such as mating success or intraspecific competition for resources, rather than absolute survival and fecundity (Agrawal & Whitlock, 2012). Defining load in terms of relative rather than absolute fitness means that the appropriate *W*_*max*_ is the fittest individual in the population, not a mutationless individual. High load then represents large differences in competitive ability among members of a population, not a threat to population survival. However, at the molecular level, many deleterious mutations simply break functionality. While the biggest immediate impact of impaired cellular metabolism might be on relative competitiveness, inferior functioning at the molecular level will inevitably also have absolute effects. While some load might be strictly relative, some will be absolute. Endlessly deteriorating relative fitness is anyway a problematic formulation of evolution.

Thirdly, load is much lower if the effects of most deleterious mutations are restricted to their impact on traits under stabilizing selection (Charlesworth, 2013). In a trait-based model, all mutations modify the value of a higher-level trait, and load is determined by the distance between this value and some optimum value. When the trait deviates far from the optimum, the fraction of mutations that are beneficial rises much higher, eventually approaching 50% in Fisher’s geometric model (Fisher, 1930). At equilibrium, this model suggests quite small loads (only about 5% for humans) (Charlesworth, 2013). But again, at the molecular level at which new mutations actually occur, a DNA change in a protein is far more likely to simply reduce general functionality than to slightly modify a higher-order trait, suggesting that unconditionally deleterious mutations represent a substantial portion, if not the vast majority, of new mutations (Karczewski et al., 2020).

Lastly, load could be cleared faster than it arises even for *U*_*d*_ > 1 if epistasis among deleterious mutations was on average synergistic (Kimura & Maruyama, 1966; Kondrashov, 1995a, 1995b). Synergistic epistasis allows one selective death to remove greater than one deleterious mutation on average, by increasing variance in fitness above that predicted in the absence of epistasis from variance in mutation number. Unfortunately, empirical data has not supported significant synergistic epistasis, suggesting that the average interaction between new deleterious mutations is close to multiplicative (Kouyos et al., 2007).

The central unresolved problem is that when *U*_*d*_ > 1, deleterious mutations fix at a higher rate than they revert (Kondrashov, 1995b), creating an endless series of deterioration. Some proportion of these mutations may affect traits under stabilizing selection or relative fitness traits, but some portion has an absolute impact, such that the system is constantly degraded.

This fundamental issue shows up in studies that attempt to model and/or infer differences in load between populations. Such studies take a variety of questionable strategies to deal with the tendency for even their large control populations to degrade. For example, some studies periodically re-normalize simulated fitness data to cosmetically remove ongoing degradation (e.g. compare Fig. S2 to Fig. 2 in (Simons et al., 2014)). Others use *U*_*d*_ < 1, e.g. (Kyriazis et al., 2021). Others treat one-locus models (Gravel, 2016; Koch & Novembre, 2017; Lohmueller et al., 2008; Simons et al., 2014)’ despite the fact that independent evolution even of unlinked sites breaks down for *U*_*d*_ > 1 (Matheson & Masel, 2023). The lack of a sound baseline model is an obstacle to reliable inference.

Indefinite deterioration can be prevented by design by using a finite sites model, but this makes load far higher than an 87% fitness reduction. Consider two alleles at each locus, one beneficial and one deleterious, with some equilibrium probability of encountering each. Sites with small selective differences will often be found in the deleterious state. When parameterized for humans, this model predicts a load with one hundred “lethal equivalents” in the exponent, prompting the expression that we should have ‘died one hundred times over’ (Kondrashov, 1995a).

When not all deleterious mutations can be purged, which ones fix will depend on their selection coefficient. A ‘drift barrier’ (Ohta, 1973; Sung et al., 2012) describes the minimum magnitude of deleterious mutation that can be reliably purged. Overwhelmingly high deleterious mutation rates will increase background selection even in the absence of linkage (Matheson & Masel, 2023), lowering the effective population size down to a point where a greater fraction of deleterious mutations will fix, including those of larger effect sizes.

Our hypothesis is that biological populations are not at equilibrium, and that nothing stops or reverts Ohta’s ratchet, i.e. the steady accumulation of slightly deleterious mutations. Instead, we hypothesize that the reason that populations persist in the face of ongoing mutational degradation is that rarer, large-effect beneficial mutations compensate for the fitness lost through many small-effect deleterious fixations. This view arises naturally from an infinite sites model with a distribution of fitness effects. Deleterious mutations with smaller *s* (Kimura, 1962) and beneficial mutations with larger *s* (Haldane, 1927) are more likely to fix. An illustrative example of this hypothesis is many proteins accumulating small deleterious mutations that slightly inhibit folding, that are compensated for by a novel or improved or overexpressed chaperone protein (Fares et al., 2002). This illustrates how, while the flux of beneficial fixations will more than cancel out the flux of deleterious fixations, this does not imply detailed balance at individual loci. Empirical evidence of this pattern of asymmetric adaptation to deleterious load has been observed in influenza (Koelle & Rasmussen, 2015), illustrating how finite sites models of detailed balance poorly describe biological populations undergoing adaptation within a vast genotype space of the possible.

Whitlock (2000) previously developed this idea, and found that populations remained stable down to a critical effective population size of barely over 100. However, this optimistic result ignored the effects of linkage disequilibrium. The flux of fixations of beneficial mutations is lower than it would be if they were evolving independently; it is reduced both by clonal interference (negative linkage disequilibrium with other beneficial mutations) (Hill & Robertson, 1966) and by background selection (positive linkage disequilibrium with deleterious mutations) (Assaf et al., 2015; Good & Desai, 2014; Pénisson et al., 2017). These same factors also cause more deleterious mutations to fix.

The full complexities of multilocus linkage disequilibrium can be captured only by simulation. Most forward time simulation methods hold a product such as *sN* constant, and rescale *N* to be smaller and *s* to be larger in order to accelerate computation (Haller & Messer, 2017). The problem with this is that it reduces the number of segregating mutations, and so understates the impact of linkage disequilibria. We instead model the evolution of load in populations with a census population size of 20,000, which we find gives rise to a realistic level of human neutral diversity (*N*_*e*_ ~7500), allowing linkage disequilibrium to emerge appropriately. We introduce two new simulation techniques to overcome the computational challenges of such an approach: ‘linkage blocks’ that avoid the need to track every single segregating site in order to perform fitness calculations, and binary indexed trees that allow both birth-death and selection processes to occur in O(log *N*) time. While linkage blocks allow us to rapidly compute individual fitnesses without real-time tracking of every mutation, we still need information about all fixed mutations at the end of the run, in order to determine the degree of asymmetry of effect sizes between fixed beneficial and deleterious mutations. To obtain this, we use tree-sequence recording (Kelleher et al., 2018), which increases our runtime substantially, while still being much faster than basing fitness calculations on individual mutations.

Our goal is to determine whether beneficial mutations are sufficient to recover fitness lost to Ohta’s ratchet in the crucial case of realistic mutation rates and linkage disequilibrium. Our metric is fitness flux, i.e. the mean rate of change in relative fitness in the population (Gravel, 2016; Mustonen & Lässig, 2010). If asymmetric adaptation is sufficient to explain population persistence in the face of accumulating deleterious mutations, then we expect to see positive fitness flux even in simulated populations with conservatively low estimates for the rates of beneficial mutations and their effect sizes. We also use our model to predict the consequences of a recent increase in the human mutation rate for human populations (Muller, 1950), and the consequences of a high deleterious mutation for variation in fitness within populations.

## Methods

Our individual-based forward-time simulations were written in C. Each individual has two characteristics: a genome, and a fitness value derived from it. Each individual’s genome is represented as two haplotypes, each an array of *L* non-recombining ‘linkage blocks’, divided into 23 chromosomes. Each linkage block consists of a floating-point variable *l*_*j*_, which summarizes the fitness effects of all mutations that occurred in the history of that linkage block, such that *l*_*j*_ = ∏_*i*_(1 +*s*_*i*_). We assume a multiplicative form of co-dominance and no epistasis, such that $w_{i}=\prod_{j=1}^{L}\left(l_{j,1}\right)\prod_{j=1}^{L}\left(l_{j,2}\right)$ where *l*_*j*,1_ and *l*_*j*,2_ refer to the effect of linkage block *j* in haplotypes 1 and 2, respectively. Note that this computationally convenient choice is not precisely equivalent to a typical codominance model, where 1 + *s*_*i*_ is the fitness of a homozygote and 1 + *s*_*i*_*h*_*i*_ is the fitness of a heterozygote. While co-dominance is unrealistic for strongly deleterious mutations, which are often highly recessive, it is reasonable for the small-effect deleterious mutations which drive Ohta’s ratchet (Agrawal & Whitlock, 2011; Simmons & Crow, 1977; Yang et al., 2017).

In addition to independent assortment of chromosomes, recombination occurs at hotspots between linkage blocks via crossing-over events between homologous chromosomes. We simulate exactly two recombination events per chromosome per meiosis, matching data for humans (Pardo-Manuel De Villena & Sapienza, 2001), although we don’t explicitly simulate a centrosome. Representing a genome as a set of ‘linkage blocks’ is a good approximation of population genetics in non-microbial species (Good et al., 2014; Neher et al., 2013; Weissman & Hallatschek, 2014). Realistic values of *L* in humans are in the range of 10^5^ −10^6^ (Altshuler et al., 2008; Belmont et al., 2005; Coop et al., 2008; Pratto et al., 2018; Wall & Pritchard, 2003). Once *L* ≥ 50 × 23 = 1150, results converge (Supplementary Figure 1), so for computational efficiency we use *L* = 50 × 23. This simplification should overestimate the effect of linkage between selected mutations, which is conservative with respect to the ability of beneficial mutations to counteract load.

Following recombination, we sample the number of new deleterious mutations in the gamete from a Poisson distribution with mean *U*_*d*_. Our distribution of fitness effects is based on a large empirical study of Europeans (Kim et al., 2017), who fitted a gamma distribution for 2*N*_*e*_*s*_*h*_ with mean −224.33, shape parameter *α* = 0.169 and scale parameter *β* = 1327.4. After drawing a value of 2*N*_*e*_*s*_*h*_ from this distribution, we rescale to using their inferred *N*_*e*_ = 11,823. We use the value drawn from this distribution as our *s*_*i*_ value. We sample the number of new beneficial mutations from a Poisson distribution with mean *U*_*b*_, and fitness effects drawn from an exponential distribution with mean (again, this is the fitness effect in the heterozygote). We explore a range of values for *U*_*b*_ and *s*_*d*_ that we consider *a priori* plausible: *U*_*b*_ ~ 0.0001–0.01 and *s*_*b*_ ~ 0.001–0.01.

We simulate a Moran model with constant population size *N*. An individual chosen uniformly at random dies each time step and is replaced by a child produced by two parents, who are chosen with probability proportional to their fitness *W*_*i*_. Each generation consists of *N* time steps. The fitnesses of the population are stored in an unsorted array — in a naïve implementation, exchanging an element to represent a birth and death would be rapid, but sampling proportional to fitness would be O(*N*). The current fastest forward-time genetic simulation tools for large population sizes (e.g. both fwdpy (Thornton, 2014, 2019)) and SLiM (Haller & Messer, 2022) preprocess cumulants each generation in a Wright-Fisher model; this speeds up sampling from the fitness array, and while the processing algorithm is O(*N*), it only needs to be performed once per generation. We instead use a binary indexed tree (Fenwick, 1994) to sample fitnesses efficiently according to the cumulative probability distribution — both updating and sampling from the tree are O(log *N*). Our scheme is expected to have similar efficiency but is intended to be useful for future expansions of this approach to absolute fitness and more complex life history models (Bertram & Masel, 2019; Matheson et al., 2023), e.g. to allow better treatment of reproductive compensation (Ober et al., 1999).

We initialize the population with mutationless individuals, then conduct a ‘burn-in’ phase during which variation increases to stable levels (Supplementary Figure 2). We end the burn-in phase 500 generations after a linear regression of the variance in fitness over the last 200 generations produces a slope less than an arbitrarily chosen low value of 0.007⁄*N* that we visually confirmed to perform well (e.g. Supplementary Figure 2). The length of the burn-in phase does not strongly depend on *N* (Supplementary Figure 3).

**Figure 1.**
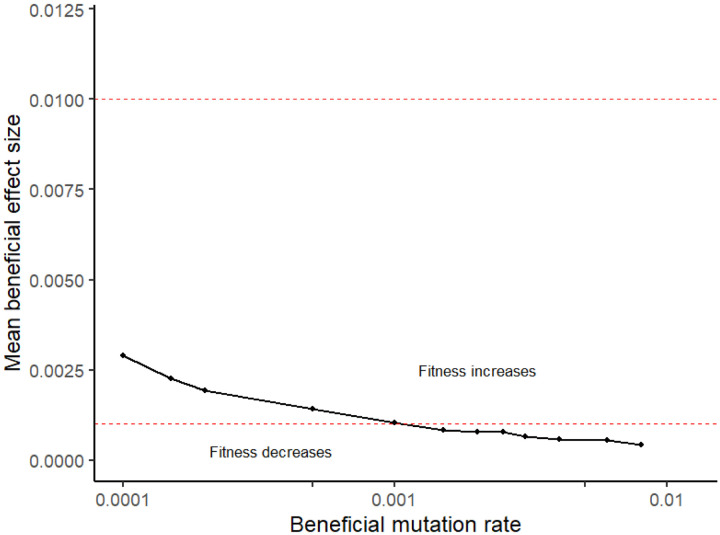
Relatively rare and mild beneficial mutations are sufficient to counteract a deluge of slightly deleterious mutations accumulating under Ohta’s ratchet. Black line shows combinations of beneficial mutation parameters that produce zero net fitness flux. All populations simulated with N = 20,000, genome-wide deleterious mutation rate of 2, and 23 chromosomes with 50 linkage blocks per chromosome. Any combinations of beneficial mutation rate and mean heterozygote effect size below the black line produce net degradation. Red dashed lines show plausible upper and lower estimates of the mean effect size of new beneficial mutations in humans that we chose *a priori*.

We calculate the net fitness flux from each simulation as the slope of the regression of log mean population fitness on time after burn-in (Supplementary Figure 2, black slope following dashed line). To numerically solve for a specified net fitness flux for Figure 1, we varied *s*_*b*_ while holding *U*_*b*_ constant. Our algorithm finds values of *s*_*b*_ that bracket the target net fitness flux, and then uses a bisection method until it finds a value of *s*_*b*_ that is within ±0.00005 of the target. In practice, there was little stochasticity in the regression slope (which averages out stochasticity in the timecourse), and so this relatively deterministic method performed well.

Although the census population size *N* is a parameter of our model, the effective population size *N*_*e*_ is not, but rather emerges over the course of a given simulation. To estimate it, we used the tree-sequence recording tools from the tskit package (Kelleher et al., 2018), and used msprime (Baumdicker et al., 2022) to retroactively add neutral mutations after each simulation. We did this only for one parameter combination involving realistically high *N*, due to the significant computational cost of this procedure; this was 23 chromosomes, 50 linkage blocks per chromosome, *N* = 20,000, *U*_*d*_ = 2, *U*_*b*_ = 0.002, and *s*_*b*_ 0.0025. These parameter values produce only a small excess of adaptation above that needed to counter Ohta’s ratchet (Figure 1). We calculate *N*_*e*_ using neutral heterozygosity under an infinite-alleles model. The choice of neutral mutation rate will not affect estimated *N*_*e*_; we arbitrarily chose 1.0 × 10^−6^ per linkage block, or 1.15 × 10^−4^ per haploid genome. This produced *N*_*e*_ ~7500, on the order of effective population sizes inferred for ancestral human populations (Tenesa et al., 2007). For comparison, similar simulations with *U*_*b*_ = 0 (i.e. with background selection alone and declining relative fitness), produce *N*_*e*_ ~16,000 (Matheson & Masel, 2023).

Tree sequence recording also tracks all non-neutral mutations, so that we can identify those that fixed and thus determine the degree of asymmetry in the effect sizes of fixed mutations. Note that without tree-sequence recording, this information would be inaccessible due to the way we summarize the fitness of many mutations within linkage blocks. However, using tree-sequence recording for all non-neutral mutations significantly increases the computation time of simulations. When we are solving for the parameters that produce a target value of net fitness flux, we therefore do not use tree sequence recording.

## Results

Achieving positive mean population fitness flux depends primarily on the mean beneficial effect size, not on the beneficial mutation rate (Figure 1), in agreement with prior theoretical work (Weissman & Barton, 2012). The black line in Figure 1 shows the parameter values for which there is exactly zero change in fitness. The entire range of *U*_*b*_ shown in Figure 1 is likely conservative, while the dashed red lines show the range for $\bar{S_{b}}$ (the mean beneficial effect in heterozygotes) that we deemed *a priori* plausible. In the absence of environmental change, population persistence is possible for $\bar{s_{b}}>\sim 0.001-0.003$, depending on assumptions about *U*_*b*_. While there is great uncertainty in the true values of these parameters, this range seems entirely plausible.

**Figure 2.**
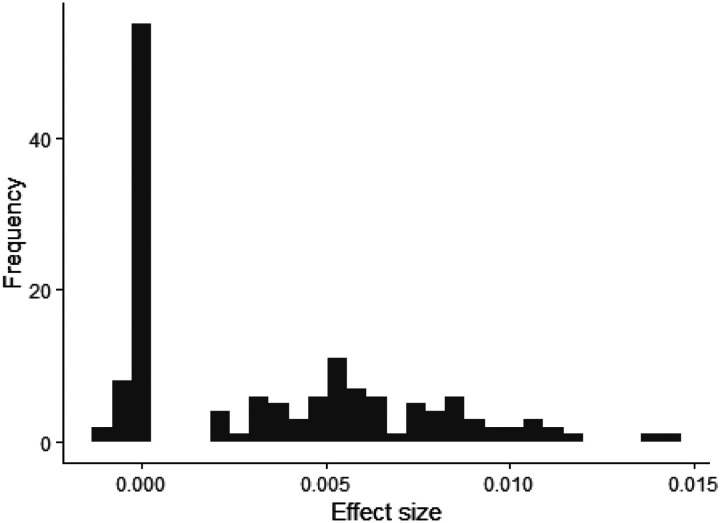
Effect sizes of fixed beneficial and deleterious mutations are strongly asymmetrical. The distribution of effect sizes of fixed mutations is shown after 5000 generations, in a population of N = 20,000 with individuals having 23 chromosomes, 50 linkage blocks per chromosome, with a beneficial mutation rate of 0.002 per generation and mean beneficial effect size of 0.0025.

The reason that such low beneficial mutation rates are sufficient for population persistence is that each beneficial mutation that fixes has a much greater magnitude selection coefficient than each deleterious mutation that fixes (Figure 2). Beneficial fixations are larger on average than new beneficial mutations, and deleterious fixations are much smaller on average than new deleterious mutations. Even in simulations that improve in fitness on average, deleterious fixations outnumber beneficial fixations.

**Figure 3.**
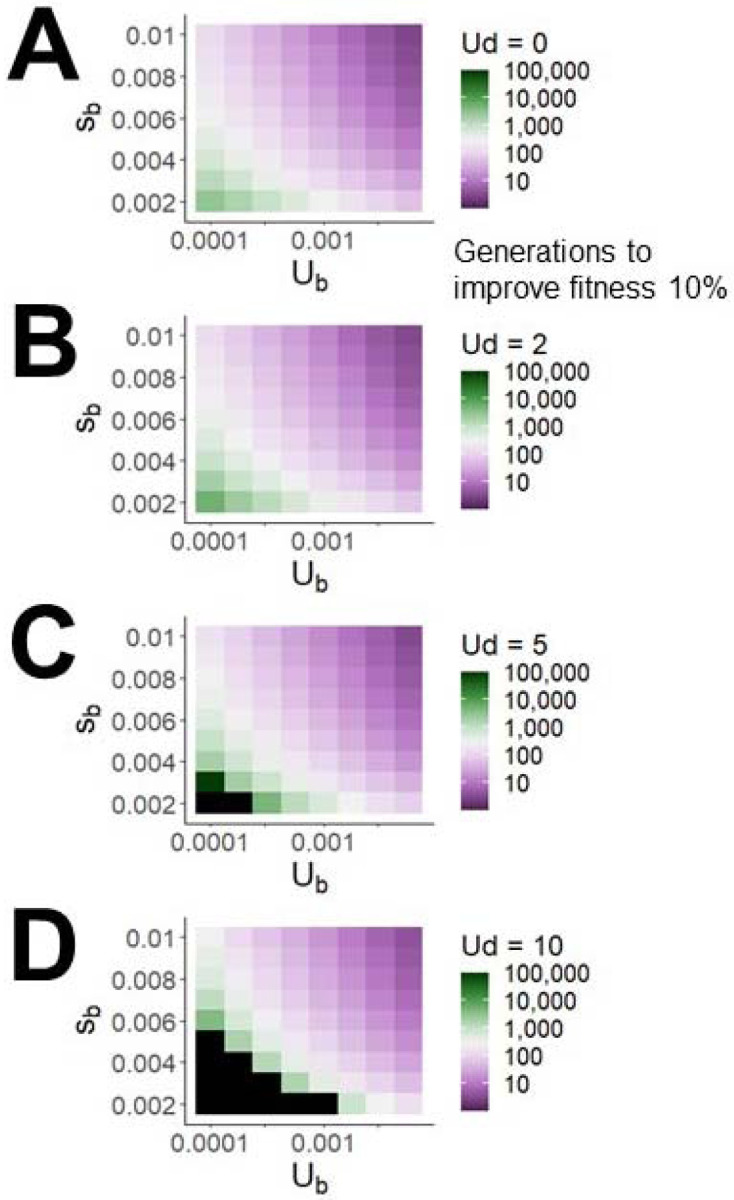
Deleterious mutations appreciably but modestly slow adaptation, visualized as the number of generations required for population mean fitness to increase by 10%. Black boxes indicate simulations with net fitness flux < 0.

We next consider the net fitness flux available for adaptation to a changing environment, above and beyond that required to counterbalance Ohta’s ratchet. Figure 3A shows how baseline (*U*_*d*_ = 0) adaptation rate depends on both *U*_*b*_ and $\bar{S_{b}}$ within our parameter value range. Figures 3B–D show how adaptation slows in the presence of *U*_*d*_ of 2, 5, and 10. Resistance to degradation remains reasonably robust, but the net fitness flux available for adaptation to a changing environment falls by ~13%, ~26%, and ~39%, respectively.

Population geneticists have raised concerns about the increase in mutation rate (Lynch, 2016; Muller, 1950), in particular due to increased age at paternity (Crow, 1997). The mean paternal age in the U.S. increased from 27.4 to 30.9 years of age between 1972 and 2015 (Khandwala et al., 2017; Kong et al., 2012), which is expected to correspond to a 12 percent increase in mutation rate. We simulated a corresponding increase the mutation rate for both deleterious and beneficial mutations of 10 percent, for a reference population with *N* = 20,000, *U*_*b*_ = 0.002, ${\bar{s}}_{b}=0.0025$ and all other parameters the same as in Figure 1. Surprisingly, populations with increased mutation rates took only 127 generations to increase their fitness by 10%, compared to 151 generations for the baseline population. In other words, because beneficial fitness flux is more sensitive to *U*_*b*_ than deleterious fitness flux is to *U*_*d*_, increasing the total mutation rate helps the population adapt faster. The counter-intuitively increased rate of adaptation directly contradicts dysgenic fears about the consequences of elevated mutation rates on mean population fitness load.

**Figure 5.**
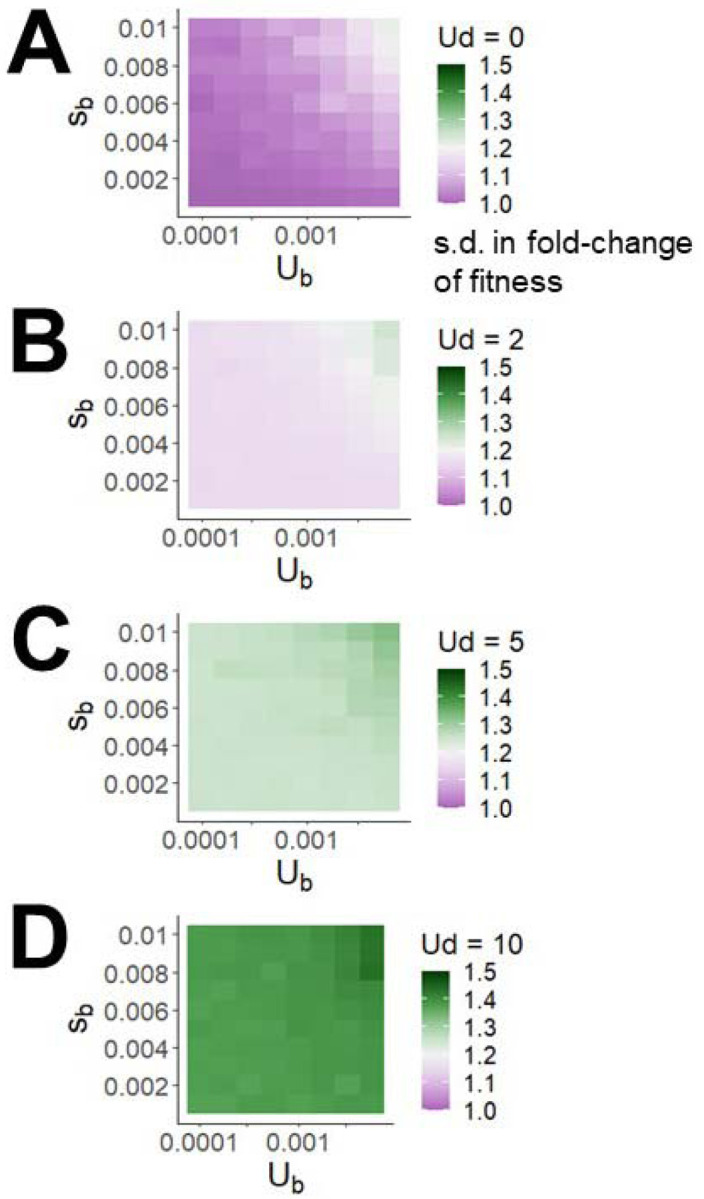
Higher deleterious mutation rates result in substantially more within-population variation in fitness at the end of a simulation, shown here as the standard deviation of fold-difference in fitness.

While high human *U*_*d*_ ~2 – 10 has only a moderate impact in reducing adaptation rate by 13–39%, its impact on variance in load among individuals within a population (Figure 5) is substantial. Perhaps unsurprisingly in light of Fisher’s Fundamental Theorem, high steady state variance in fitness among individuals within a population seems to be an inevitable consequence of high *U*_*d*_. With fitness being log-normally distributed, Figure 5 expresses this variance in terms of the fold-difference between two individuals that are one standard deviation apart. This variance is relatively insensitive to *s*_*b*_ and *U*_*b*_, but depends dramatically on *U*_*d*_. Figure 5 suggests that differences in deleterious load cause two randomly sampled humans to have a typical difference in fitness (in the historical human environment) of 15%−40%. Beneficial parameters have little effect on within-population variation in fitness, increasing it mostly only at the highest beneficial mutation rates and mean effect sizes we consider.

## Discussion

We address the puzzle of how populations persist given the threat posed by realistically high deleterious mutation rates. Unlike many previous solutions, we allow that many slightly deleterious mutations do in fact accumulate (Ohta’s ratchet), but argue that this does not lead to population deterioration because a smaller number of beneficial fixations of greater size successfully counteracts many more small-effect deleterious fixations. We demonstrate the plausibility of this asymmetric compensation scenario under realistic values for deleterious mutation rate and sizes, recombination rate, and beneficial mutation size, and conservative values for the beneficial mutation rate. While population persistence is achieved, the need to counterbalance deleterious mutations does exact an appreciable toll in terms of a 13–39% reduction in the speed of adaptation to a changing environment. Our model of realistic deleterious mutation rates logically entails high variance in fitness (in ancestral environments) within human populations.

While our explanation for population viability requires only conservatively low beneficial mutation rates, detailed balance would require much higher *U*_*b*_. E.g. in an asexual model with *U*_*d*_ = 2, *s* = 0.01, and *N* = 10,000, an analytic approximation suggests that more than 30% of new non-neutral mutations would need to be beneficial to counteract deleterious load (Goyal et al., 2012), which is implausibly high. As reviewed in the Introduction, solutions that ignore the fundamentally damaging nature of mutations at the molecular level, e.g. to focus instead on quantitative traits, involve unrealistically high beneficial mutation rates.

Synergistic epistasis has often been invoked as the solution to mutation load and its accumulation, but most models invoke a quantitatively extreme form of synergistic epistasis, truncation selection (Crow & Kimura, 1979; Kondrashov, 1982). However, empirical assays of *de novo* deleterious mutations in bacteria and eukaryotic microbes do not show any synergistic epistasis on average (Elena & Lenski, 1997; Kouyos et al., 2007), let alone truncation selection. Worse, mutation accumulation experiments often show decreases in the rate of decay of fitness, consistent with antagonistic epistasis among deleterious mutations (Francisca & F., 2007; Maisnier-Patin et al., 2005; Perfeito et al., 2014). On the other hand, experimental evolution studies consistently find diminishing returns epistasis (which corresponds to synergistic epistasis if viewed from the perspective of deleterious mutations) between new beneficial mutations (e.g. (Barrick et al., 2009)). These apparently contradictory observations can be reconciled in multiple ways. One hypothesis is that mutations have massively multidimensional interactions across the genome and a given mutation’s fitness effects are uncorrelated across interactions (idiosyncratic epistasis (Lyons et al., 2020)). Another hypothesis is that mutational effects are multiplicative (or antagonistic or idiosyncratic) between functional modules, while being synergistic within modules (Rice, 1998; Wei & Zhang, 2019). Theory has not yet been developed to show whether these more nuanced forms of epistasis, compatible with data, could purge mutation load fast enough to avoid population degradation.

Sexual selection might also assist with purging load (Grieshop et al., 2021; Whitlock & Agrawal, 2009). While human monogamy reduces the scope for sexual selection, increased variance in fitness caused by assortative mating under mutual mate choice might still help prevent mutational degradation (Hooper & Miller, 2008; Kvarnemo, 2018).

Our hypothesis of asymmetric deleterious and beneficial fixations parallels known features of molecular adaptation. For examples, many mutations that each jeopardize the stable folding of a protein can be ameliorated at once by the evolution of chaperones (Fares et al., 2002; Gros & Tenaillon, 2009). Many poorly splicing introns can be ameliorated by the evolution of a better spliceosome (Wu & Hurst, 2015).

**Figure 6.**
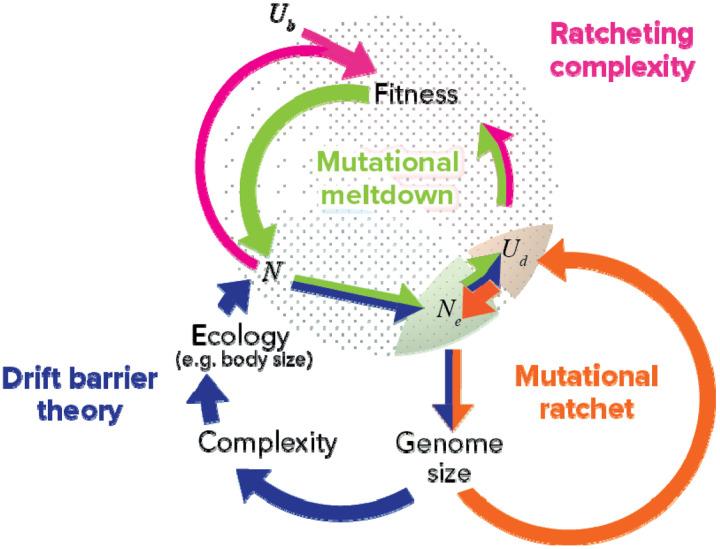
A feedback loop of ratcheting complexity can be driven either by census size and ecology (drift barrier theory, blue) or by high deleterious mutation rate (our view, orange and pink). The drift barrier ratchet requires low census population size, whereas our ratchet requires high deleterious mutation rate. Drift barrier theory emphasizes a causal link from to via relaxed selection against mutators (Lynch, 2007), whereas we emphasize background selection as a causal driver in the opposite direction, i.e. from *U*_*d*_ to *N*_*e*_. Mutational meltdown (Lande, 1994; Lynch et al., 1995) is shown for completeness (green), since most of its elements are already invoked.

A pattern of many small mutations, each of which cannot be effectively cleared, being counteracted by compensatory mutations with global effects, has previously been predicted by drift barrier theory (Fares et al., 2002; Frank, 2007; Gros & Tenaillon, 2009; Lynch, 2007, 2010a; Rajon & Masel, 2011; Sung et al., 2012; Wu & Hurst, 2015; Xiong et al., 2017). Drift barrier theory, as put forward by Lynch (2007), is illustrated in blue in Figure 6. Drift barrier theory emphasizes the causal importance of census population size in producing a ratcheting effect that leads to increased molecular and organismal complexity. Effective population size (with respect to the minimum size of a deleterious mutation that can be reliably purged) is posited to be driven (albeit not exclusively) by census population size, which is in turn driven by life history traits such as body size (Lynch, 2007, see Chapter 4). A low effective population size that cannot purge small DNA insertions leads to a bloated genome, whose complexity is posited to lead to larger body size and/or increased ecological specialization, reducing census population size, which closes the causal loop. Increased mutation rate is seen primarily as a consequence of relaxed selection against mutator alleles.

Our results suggest a shift in perspective, placing causal emphasis on a high deleterious mutation rate instead of on a low census population size. Indeed, a mutational ratchet cycle (Figure 6, orange) can occur even when census population size is high. First, a sufficiently high deleterious mutation rate accelerates Ohta’s ratchet (the inevitable accumulation of slightly deleterious mutations), because background selection (among unlinked sites) substantially reduces *N*_*e*_ once *U*_*d*_ > 1 (Charlesworth, 2012; Matheson & Masel, 2023). As with drift barrier theory, the resulting deluge of slightly deleterious fixations increases genome size, but the feedback loop from there does not go through census *N*. Instead, larger genomes create a larger target size for deleterious mutations, directly increasing *U*_*d*_.

The mutational ratchet described above (Figure 6 orange) drives *U*_*d*_ up to a high enough level to power the complexity ratchet (Figure 6, pink) that is the focus of this manuscript. Similarly to drift barrier theory, molecular complexity ratchets up when slightly deleterious mutations cannot be purged or reversed in a manner that achieves detailed balance, but must instead be compensated for by large effect changes that frequently occur at a higher level of organization. However, our view in Figure 6 (orange and pink) bypasses the census population size and ecological factors that are central to the drift barrier view (blue). This difference is made clear by the conditions required for each view. The drift barrier view requires low *N* but can occur at low *U*_*d*_ so long as *sN*_*e*_ is low. Our view requires *U*_*d*_ > 1 and can occur even for high census *N*.

Only some populations, like bacteria, are able to achieve a detailed balance solution to load problems that enables them to retain simple, efficient genomes. Previous hypotheses have focused on the size of such populations as the crucial divider between species that are able to purge load within a small, simple genome vs. species forced into ratcheting molecular complexity in search of innovative molecular solutions to stay ahead of perpetual degradation. But large bacterial populations also have deleterious mutation rates below 1, which provides an alternative explanation as to how they maintain streamlined genomes. The pressure of mutation load might therefore be a primary driver behind molecular complexity across the entire tree of life.

Understanding how mutation load might be stabilized in humans and similar species is a precondition for addressing a long-standing concern of geneticists: that load might be increasing in modern humans because of recent changes to human lifestyles or technology. For example, if mutation rate, beginning already at a critically high level, increases further due to increased paternal age, or if selection against deleterious mutations is relaxed due to modern medicine, the perception has been that load should increase, potentially with disastrous consequences (Crow, 1997; Lynch, 2016). Intriguingly, our results suggest that the approximate increase in mutation rates in human populations due to increased paternal age have the opposite effect, improving rather than degrading population mean fitness.

However, high *U*_*d*_ has profound consequences for understanding within-population differences among individuals. While a genotype whose load used to cause a ~30% reduction in fitness in ancient human environments might now have a lesser impact on fitness, it likely still has a significant impact on health. Indeed, variation in self-reported health has a substantial genetic component (Romeis et al., 2000), and load, as assessable from whole-genome sequencing, can be used to predict medically relevant phenotypes (Fiziev et al., 2023; Vy et al., 2021). High genetic variance among individuals is a hidden confounding variable in a vast range of studies (Harden, 2021), including many studies of human health. Our theoretical assessment implies necessarily high variance in human mutation load. This should trigger a significant reassessment across all public health studies grounded in correlational analysis (Harden, 2021).

We have shown that populations are able to survive the constant accumulation of mildly deleterious mutations (Ohta’s ratchet) by acquiring a smaller number of larger-effect beneficial mutations. Mutation load may therefore not threaten population persistence, but this does still suggest that load is a crucial evolutionary factor with diverse effects. These include driving the evolution of molecular and organismal complexity, and maintaining high rates of fitness variance within populations.

## Supplementary Material

Supplement 1
