# Supplementary material for: Human deleterious mutation rate implies high fitness variance, with declining mean fitness compensated by rarer beneficial mutations of larger effect
Source: bioRxiv. 2023 Sep 4:2023.09.01.555871. Preprint. [Version 1] doi: 10.1101/2023.09.01.555871 (PMC10508744; doi:10.1101/2023.09.01.555871)
Supplement: Supplementary file 1 [file media-1.docx]

**Supplementary Figure 1.** Dividing each of our 23 chromosomes into fewer than 50 linkage blocks would substantially underestimate adaptation, but using more linkage blocks has only small benefits. All have $N=20,000$, $U_{d}=2$, $U_{b}=0.001$, and mean $s_{b}=0.005$.

**Supplementary Figure 2. The burn-in phase ends once variance in fitness plateaus.** Dashed line indicates the end of the burn-in phase, at generation 1227. The slope of log mean fitness (black) after the burn-in phase describes the net fitness flux in the population over time. N = 20,000, each individual has 23 chromosomes with 50 linkage blocks per chromosome, $U_{b}=$0.002, and $s_{b}=$ 0.0025.

**Supplementary Figure 3.** The number of generations until the end of the burn-in phase increases slowly with increasing population size.
